# Production of Platinum Atom Nanoclusters at One End of Helical Plant Viruses

**DOI:** 10.1155/2013/746796

**Published:** 2013-09-25

**Authors:** Yuri Drygin, Olga Kondakova, Joseph Atabekov

**Affiliations:** ^1^Belozersky Institute of Physico-Chemical Biology, Lomonosov Moscow State University, Vorob'evy gory 1, Building 40, Moscow 119992, Russia; ^2^Department of Biology, Lomonosov Moscow State University, Vorob'evy gory 1, Building 12, Moscow 119992, Russia

## Abstract

Platinum atom clusters (Pt nanoparticles, Pt-NPs) were produced selectively at one end of helical plant viruses, tobacco mosaic virus (TMV) and potato virus X (PVX), when platinum coordinate compounds were reduced chemically by borohydrides. Size of the platinum NPs depends on conditions of the electroless deposition of platinum atoms on the virus. Results suggest that the Pt-NPs are bound concurrently to the terminal protein subunits and the 5′ end of encapsidated TMV RNA. Thus, a special structure of tobacco mosaic virus and potato X virus particles with nanoparticles of platinum, which looks like a push-pin with platinum head and virus needle, was obtained. Similar results were obtained with ultrasonically fragmented TMV particles. By contrast, the Pt-NPs fully filled the central axial hole of *in vitro* assembled RNA-free TMV-like particles. We believe that the results presented here will be valuable in the fundamental understanding of interaction of viral platforms with ionic metals and in a mechanism of nanoparticles formation.

## 1. Introduction 

One current goal in nanobiotechnology is to investigate biological nanoplatforms capable of binding metal atoms with the aim of generating new bioinorganic materials for nanoelectronics and medicine. Helical plant viruses, in particular tobacco mosaic virus (TMV) and potato virus X (PVX), have been widely used as templates and scaffolds in nanotechnology [1–12]. 

TMV particles are rod-like with a diameter of 18 nm and modal length of 300 nm. They consist of 2130 identical 17.5 kDa protein subunits helically arranged around a cylindrical canal and closely packed into a rigid tube. The two-layer cylindrical substructure, each layer consisting of a ring of 17 molecules of coat protein (CP), is known as a “disk,” and 16 1/3 molecules are present in each turn of the assembled helix. RNA is introduced between the CP turns and follows the helix of protein subunits [13, 14]. Apparently, the front stereochemical surface of terminal CP molecules of the helical TMV particle is not equivalent to inner surfaces of other CP subunits.

It is known that in the absence of RNA the viral CP may be *in vitro* assembled into several types of aggregate. In particular, TMV CP can be *in vitro* assembled into virus-like particles (VLPs) that are structurally similar to native virions [15, 16].

The virions of another helical virus, PVX, are flexuous filaments with modal lengths in the range of 470–580 nm and a diameter of approximately 13 nm [17].

 In this work, we found that ions of platinum coordinate compounds reduced by borohydrides could form nucleation centers for the very selective growth of nanoparticles on one pole of helical plant viruses (tobacco mosaic virus and potato virus X). 

## 2. Materials and Methods 

### 2.1. Modification of Viruses with (Dien)platinum, (Chloro(diethylenetriamine)platinum(II) Chloride or [Pt(dien)Cl]Cl), and Sodium Borohydride

100 *μ*L of TMV or PVX suspension (0.4 mg/mL) in TDW (deionized double distilled water) was mixed with 2 *μ*L of 25 mM [Pt(dien)Cl]Cl in TDW. The mixture was incubated for 1 h at 30°C, then borate buffer 0.5 M (pH 8.3) was added up to 0.1 M. The reaction mixture was cooled in an ice bath, and sodium borohydride (5mg/mL) was added portion wise to a final concentration of 3 mM or 10 mM. To do Pt-NPs larger and produce short nanowires, concentration of (dien)platinum was increased two fold. The reaction mixture was diluted 4-fold with TDW and samples were examined with transmission electron microscopes Zeis LEO912 AB OMEGA supplied with the energy filter or with TEM Jeol JEM 1011.

### 2.2. Modification of TMV with (Dien)platinum and Hypophosphite

100 *μ*L of suspension of virus (TMV, PVX, 1 mg/mL) was mixed with 4 *μ*L 20 mM (dien)platinum in water (pH~7), kept for 60 min at 30°C. Then reducing agent sodium hypophosphite (Fluka) was added to a final concentration of 30 mM, and the mixture was incubated for 20 min at room temperature.

### 2.3. Electron Microscopy of the Virus RNA and Ribonucleoproteins

2% collodion solution (Fluka) strengthened by an evaporated carbon was used for preparation of support films specimens for electron microscopy. Usually, 5 *μ*L of TMV suspension was placed on the grid for 1 min, after which the droplet was removed by a filter paper. While samples were examined with transmission electron microscopes Zeis LEO912 AB OMEGA supplied with the energy filter, no staining was done. In some cases (indicated), virus particles were briefly stained with 2% uranyl acetate for additional positive contrasting. 

To observe the RNA, samples were prepared by the protein-free monolayer spreading method described in [18]. Concentrations in the spreading solution were 0.1 mg/mL TMV, 3 M urea, 3.7% formaldehyde, and 0.01% benzyldimethylalkylammonium chloride. The hypophase was 0.15 M sodium acetate and 0.1% formaldehyde. The monolayer was picked up on collodion support, and the sample was rotatory shadowed with tungsten under 7°.

## 3. Results and Discussion

### 3.1. Polar Electroless Deposition of Platinum onto Helical Viruses

In the present work, the structure of bioinorganic nanocomplexes, produced by platinum atoms reduced on a platform of helical plant viruses (TMV, PVX) and on RNA-free TMV VLPs, was studied.

Previously, to synthesize DNA [19] and RNA [20] probes, we used the platinum coordinate compound chloro(diethylenetriamine)platinum(II) chloride (mw 369.15) as a label. [Pt(dien)Cl]Cl was synthesized according to Watt and Cude [21]. It is known that at a ratio of 1 and less per 10 nucleotide residues of double-stranded DNA, (dien)platinum is associated almost exclusively with guanine residues at position N7 [22].

Because it was possible that the (dien)platinum would react with the TMV protein, it was necessary to determine the working concentrations of the reactants and the optimal (dien)platinum/virus ratio to obtain stable complexes and avoid TMV aggregation. As found by sedimentation analysis in an analytical ultracentrifuge, the optimal ratio of (dien)platinum to the virus (in moles of CP) must be 50 : 1 or less to obtain stable (dien)platinum reacted TMV particles in solution.

**Figure 1:**
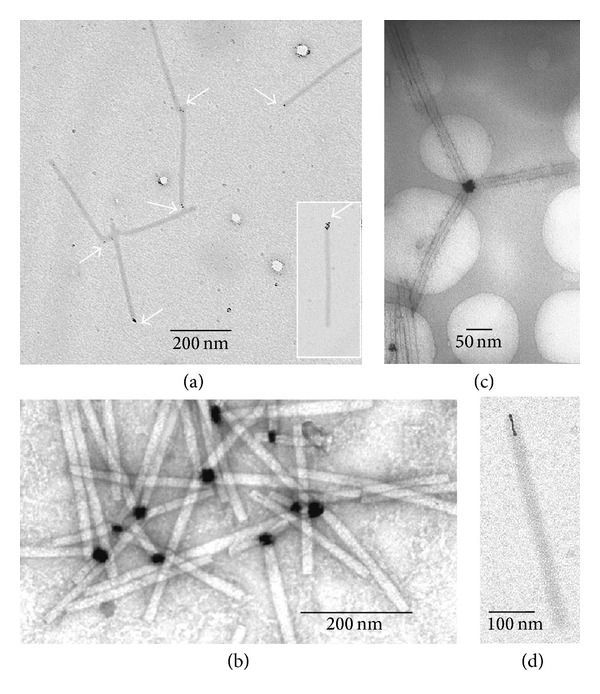
Polar growth of the platinum atom clusters on the TMV platform. Platinum nanoparticles were formed after chemical reduction of (dien)platinum by sodium borohydride to a final concentration of 3 mM (a) or 10 mM ((b)–(d)), see Section 2). Samples were examined with transmission electron microscopes Zeis LEO912 AB OMEGA supplied with the energy filter (specimens ((a), (d)) and Jeol JEM 1011 (specimens (b), (c)). No staining of specimens ((a), (d)) was carried out. Samples ((b), (c)) were stained slightly with 2% uranyl acetate. Arrows in (a) indicate the platinum atom clusters bound to the end of the virus particles.

Chemical reduction of the complex platinum ions bound to the TMV platform was performed in aqueous sodium borohydride or dimethylamine borane solutions using different reaction times and excesses of the reducing agent. Clusters of platinum atoms and short nanowires formed on the native TMV scaffold were revealed under these experimental conditions (Figure 1) by transmission electron microscopy (TEM). 

Figure 1(a) shows that small (~1-2 nm) Pt nanoparticles (Pt-NPs) could be formed on one end of the TMV particles upon exposure to 0.5 mM [Pt(dien)Cl]Cl in 3 mM solution of the reducing agent at pH~8. It is noteworthy that 55% of TMV particles (200 particles were analyzed) contained the Pt nanoparticles on one end, while the number of virus particles carrying Pt-NP clusters on both ends was negligible (less than 0.5%), and no lateral electroless deposition of the platinum on the virus exterior was detected. The formation of Pt-NPs was also observed on one end of the end-to-end aggregates of TMV particles (longer than 300 nm) and TMV fragments shorter than 300 nm. Figure 1(a) (inset) shows that several small discrete Pt-NPs could also be linked to one end of the TMV particle; presumably the primary platinum nanoparticle works here as a nucleation center.

Increasing concentrations of (dien)platinum to 1 mM and borohydride to 10 mM resulted in an increase in the size of Pt-NPs to ≤30 nm as shown in Figures 1(b) and 1(c). Frequently, these TMV particles are aggregated by their terminal “Pt-NP ends” into star-like structures (Figure 1(c)). TMV particles with the central hole partly filled with Pt-nanowire (Figure 1(d)) were also revealed, comprising 20% of the total number of the virus particles. The Pt-nanowires were 10–30 nm in length with a diameter of approximately 3 nm and resembled a small helix.

**Figure 2:**
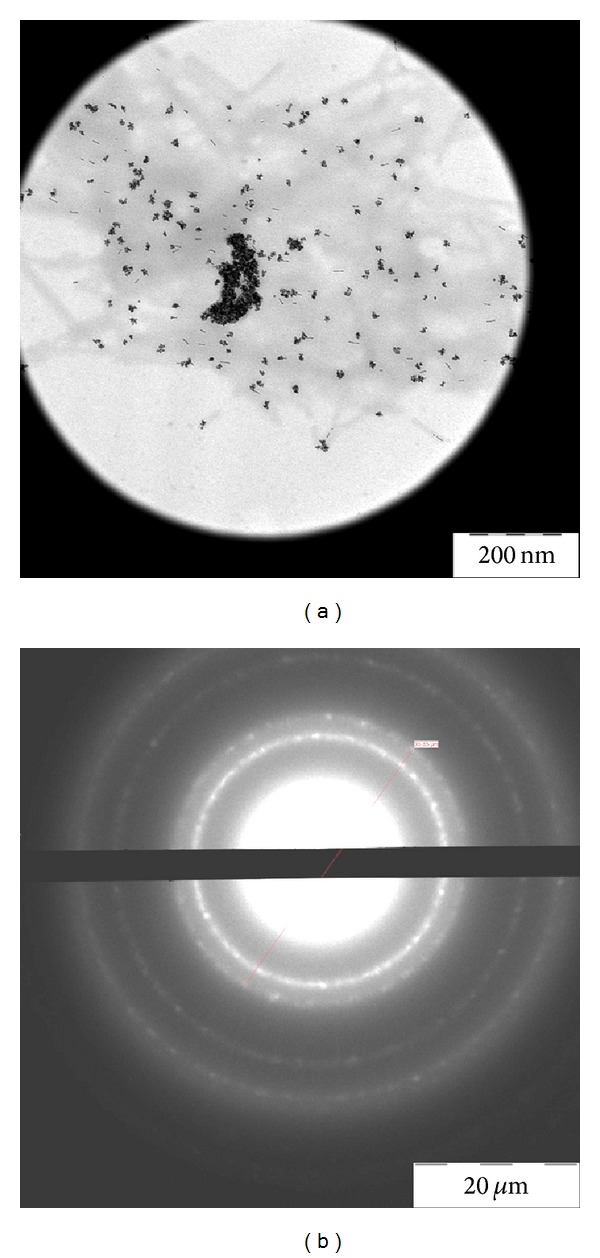
Electron diffraction of the Pt-NPs-TMV preparation. Large area image of the sample in Figure 1(b) concentrated on the specimen grid and its electron diffraction ((a) and (b), resp.).

To prove the platinum origin of the nanoparticles, solution of TMV with bound platinum nanoparticles was concentrated, and diffraction of electron on nanoparticles was examined. Diffraction of electrons for concentrated sample, shown in Figure 1(b) and unstained with uranyl acetate, was obtained by energy-filtering TEM and showed typical picture of face-centered cubic lattice of platinum (Figure 2), with intrinsic interplanar distances within the measurement error.

Production of Pt-NPs by reduction of the (dien)platinum-pretreated TMV with dimethylamine borane (10 mM, pH 7, 60 min, r.t.) was less efficient than that with sodium borohydride. Presumably, this was because of dimethylamine borane's weaker reducing potential [23].

While potassium tetrachloroplatinate (IV) was reduced by sodium borohydride in weakly alkaline conditions, polar growth of clusters of platinum atoms on virus particles was observed. One hundred of the TMV particles were examined, 55% had platinum atom clusters at one end and 3% at both ends (not shown).

**Figure 3:**
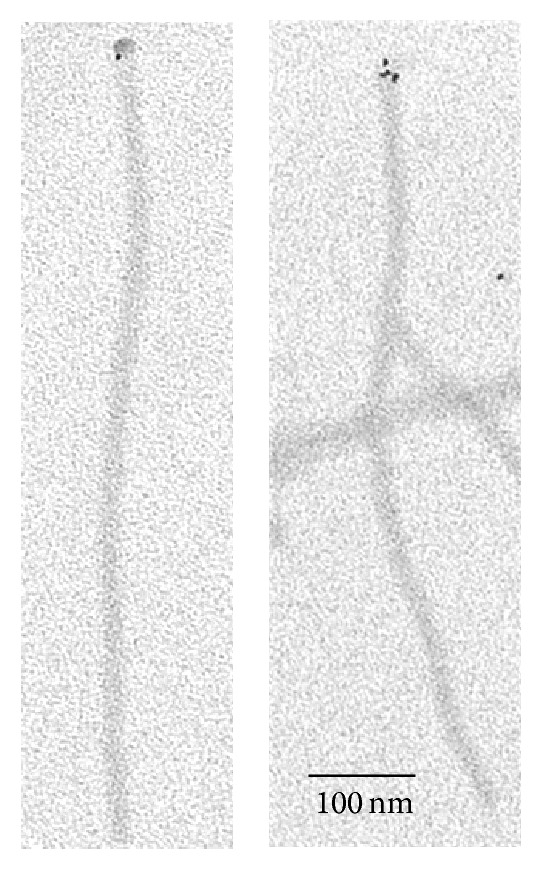
Polar binding of the platinum atom clusters to PVX particles. Sample preparation described in Section 2. Specimen was examined under TEM Zeis LEO912 AB OMEGA microscope, no contrasting of specimen.

To further elucidate this phenomenon, we examined the interaction of (dien)platinum and its reduction by sodium borohydride with another helical virus, the potexvirus.  Figure 3 shows that several discrete small Pt-NPs were associated with one end of filamentous PVX, suggesting that the polar binding of Pt-NPs to helical viruses is a common phenomenon. 

The (dien)platinum reacts in preference with guanine residues of DNA [22] and, presumably, of RNA [20]. It is known that TMV and PVX RNAs contain only two identical terminal chemical structures, both at the 5′ end, which are guanine residues in the cap group (m^7^GpppGp) [24, 25].

It appears that the recognition of one end of the TMV (and PVX) particle by Pt atoms is due to identical 5′-end cap group and peculiarities of the polar geometry of the viral helix. By its selectivity this phenomenon is similar to the specific interaction of monoclonal antibodies with antigenically different terminal surfaces at the virus particle ends [26, 27]. 

There were three possible mechanisms for platinum atom interaction with the 5′ end of the initial TMV and PVX particles: (i) (dien)platinum interacts with the RNA cap structure and Pt-NPs interact strongly with the 5′ end of the virus RNAs; (ii) (dien)platinum atoms and Pt-NPs recognize and interact strongly with the exposed surface exterior of the 5′-terminal protein or with a disk face structure; (iii) both interaction types of (dien)platinum and Pt-NPs are realized. To select between these mechanisms, the following experiments were carried out.

### 3.2. Pt-NPs Are Deposited on the 5′ End of TMV RNA

It has been shown that the stripping of TMV CP subunits from the 5′ end of RNA is induced by urea, DMSO (dimethyl sulfoxide), alkali, and detergent [28, 29]. Polar stripping results in the production of rods with a tail of RNA protruding from one end. The 5′-proximal 69 nucleotides of TMV RNA lack guanine bases and interact more weakly with CP subunits compared to other regions of RNA [30]. As a consequence, polar 5′ to 3′ uncoating of RNA occurs because of the removal of the terminal CP molecules. 

**Figure 4:**
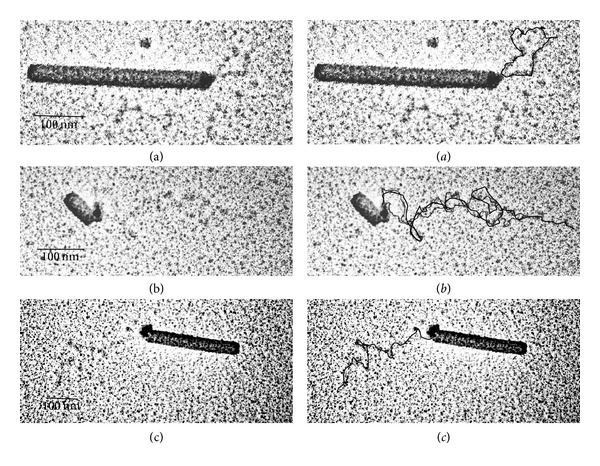
Partial uncoating of TMV and Pt-NPs complexes by 6 M urea. Uncoating was allowed to proceed at 0°C for 5 min in 6 M urea and 60 mM sodium phosphate at a virus concentration of 0.5 mg/mL. Shadowed samples (see Section 2) were examined with a Jeol JEM1011 microscope. Analysis was conducted on 130 uncoated partially TMV particles. Left: ((a), (b)) show a loop of the free RNA released from TMV particle retained by Pt nanoparticle and (c) shows the partially hydrolyzed linear RNA. It is important to note that RNA has secondary structure under spreading conditions. Right: ((*a*)–(*c*)) merge images with inked over RNA strand.

Therefore, (dien)platinum was reduced by sodium borohydride on the tobacco mosaic virus, and the Pt-NPs-containing virus particles were then treated with urea. To fix the structure obtained, formaldehyde was added up to 3.7%. Partly stripped TMV rods with a tail of RNA protruding from one end were produced (Figure 4). 

Treatment of the Pt-NPs-TMV complex with urea leads to a partial stripping of the viral capsid proteins adjacent to the nanoparticle and to the protruding free RNA (Figure 4(c)). The Pt-NPs were still associated with the shortened virus rods after removal of RNA tails by RNase A (20 *μ*g/mL, 30 min, 30°C) (data not shown). However, Figure 4 shows that, after treatment with ultrasound and urea, the Pt-NP is located on the inner edge of the truncated viral particles. How does this translocation of nanoparticles take place? 

There are two possible explanations.

(1) Since the disassembly and reassembly of TMV are reversible, and the viral proteins have a high propensity toward association, the 5′-end complex of “Pt-NPs-RNA-CP” does not move out and retain contact with the 5′ end of the truncated viral particle via protein-protein interactions. Freed bare RNA is looped out, but bound continuously to the 5′-terminal facing of the viral capsid (Figures 4(a) and 4(b)). It is well known that the purified TMV preparation has some sticky ribonuclease [31]. Free denatured RNA undergoes simple hydrolysis, producing free ends retaining links with the truncated virus particle and leaving Pt-NPs on the edge of the shortened virus rod (Figure 4(c)). 

(2) Pt-NPs bind more strongly to the 5′-terminal end of TMV preeminently because of the more appropriate spatial arrangement of the complex-forming groups of the virus protein and RNA. Therefore, the nanoparticle translocates from the disembodied capsid protein to the truncated (via the urea) end of the viral particle because of linking with bare RNA is weaker (Figure 4(c)).

This observation allows us to hypothesize that the cap groups were not accessible or weakly associated with the Pt-NPs being encapsidated in the virus helix. This also suggests that the Pt-NPs were linked tightly to CP subunits within the 5′-terminal facing or several exterior surfaces of subunits in the TMV helix. If so, the Pt-NPs were linked to the domain(s) of CP subunits located at the terminal surface and adjacent to the central hole of the helical particle. This suggestion is also in line with the ability of Pt-NPs to penetrate the internal canal of TMV tubes, producing nanowires.

### 3.3. Pt-NPs Are Bound to the 5′-Terminal Protein Facing of the TMV Virus Particle

Strong binding of the Pt-NPs to the TMV was confirmed by the linkage resistance to the ultrasonication treatment. Results of two series of experiments illustrate this finding.

**Figure 5:**
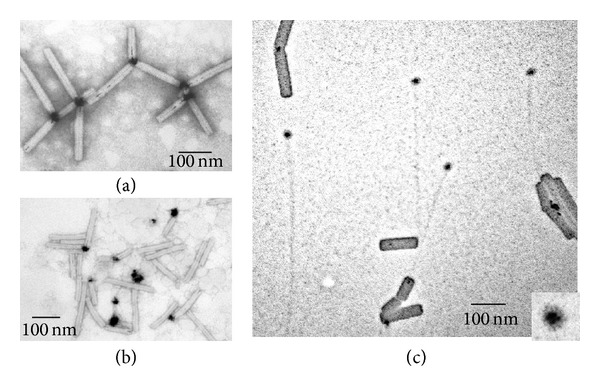
Platinum modification of the ultrasonically treated TMV. (a) chilled TMV samples (1 mg/mL, in ice water) were ultrasonicated at 40 W for five seconds six times with intervals of 60 s using the ultrasonic disintegrator MSE Soniprep 150. TMV was then reacted with 1 mM (dien)platinum and reduced by 10 mM sodium borohydride, (pH~8). (b) TMV was first incubated with 1 mM (dien)platinum and reduced with 10 mM sodium borohydride, then, was exposed to 40 W sonication for 5 s six times with intervals of 60 s at 0°C. Samples (a) and (b) were diluted 10-fold with TDW and stained with 2% uranyl acetate. To observe viral RNA (c), a sample prepared as described in (b) was developed via the monolayer technique (see Section 2). Inset shows 3-fold magnification of the Pt nanoparticle apparently included in the terminal TMV protein facing or disk.

(i) The virus was first ultrasonicated at 0°C to produce halves of particles and then reacted with (dien)platinum under the same conditions. The size of fragments varied from 25 to 200 nm with the peak (42%) at 100–120 nm. As expected, approximately half (55%, 100 TMV particles were analyzed) of sonication-generated fragments contained Pt-NPs bound to one end (Figures 5(a) and 5(b)).

(ii) TMV was first reacted with (dien)platinum and then ultrasonicated. TMV fragments ranged in size from 25 to 200 nm with two peaks: 160 nm (22%) and 120 nm (17%). Only 23% of the TMV particles (100 TMV particles were analyzed) were associated with Pt-NPs; approximately half of the Pt-NPs were detached from the virus rods (20–30% at least, some aggregated) (Figure 5(b)). One can propose that the essential part of free Pt-NPs is just shaken off from the TMV fragments by ultrasonication or they are associated with a low contrasted and weakly visible filamentary material (RNA?).

To gain further insight into the origin of a low contrasted filamentary material with apparently bound Pt-NPs, the fragments generated by sonication of reacted with (dien)platinum virus were fixed with formaldehyde, and benzylalcylammonium chloride was then added. To spread over RNA molecules, urea was added up to a final concentration of 3 M, and the reaction cocktail was loaded onto a hypophase (0.15 M sodium acetate and 0.1% formaldehyde). 

Significantly, this technique revealed unusual structures comprising the disk-like element (apparently consisting of CP subunits) with Pt-NPs bound to this disk and the tail of RNA connecting the disk with the remainder of the stripped TMV particle (Figure 5(c) and inset). We assume also that the Pt-NP, by binding to the 5′ end of the RNA and to the 5′-terminal protein (or disk) facing of the TMV virus particle, weakens the connection between this exterior and the rest of the virus particle. 

The results obtained suggest that the rigid protein shell is broken into fragments by sonication, while the flexible RNA remains intact (Figures 4 and 5(c)). This finding can be explained by the varying diametrical stiffness of the virion and RNA. It should be stressed that uncoating the TMV modified with (dien)platinum by ultrasonic treatment starts from one end (Figure 4) and is similar to the native TMV uncoating with urea, DMSO, alkali, SDS (sodium dodecyl sulfate), and heat treatment [28, 29]. This confirms, despite its seemingly symmetrical form (see, e.g., the PDB 3D image for TMV), that the virus particle has chemical and physical structural asymmetries at input and output ends. Interestingly, this is logically related in the biological sense to the polarity of TMV RNA-concurrent with uncoating, translation of the parent virion RNA starts at the 5′ end [30]. 

It appears that the platinum nanoparticle is bound both to the 5′ end of the viral RNA and to the capsid protein, and this association is resistant to the ultrasonic processing and urea treatment. Taken together, these data provide strong evidence that Pt-NPs can be linked directly to the first 5′ surface exterioir of terminal subunits in the TMV CP helix. Concurrent link formation between the Pt-NPs and the 5′ end of encapsidated RNA is also indicated. 

It is difficult now to predict with which atoms of amino acid residues of CP and nucleotide residues of RNA at the very 5′-end of the virus RNA interacts (dien)platinum ion without special study of (dien)platinum docking on the pole TMV and PVX exterior surfaces. In connection with this, it would be interesting to add other ligands to a platinum modified virus particle that are able to enter the metal coordinate sphere, using chemical properties of coordinate metals and clusters of their atoms bound to a virus.

### 3.4. Platinum Modification of TMV-Like Particles

As mentioned above, RNA-free TMV-like particles can be assembled from viral CP *in vitro* [15–17]. 

CP was extracted from TMV using an acetic acid according to Fraenkel-Conrat et al. [32]. Then, a sodium phosphate buffer (100 mM, pH 5.6) was added to the suspension of TMV CP to obtain a final concentration of 1 mg/mL, and the protein was incubated at room temperature for 24 h in order to produce VLP. This solution was stored at 4°C.

**Figure 6:**
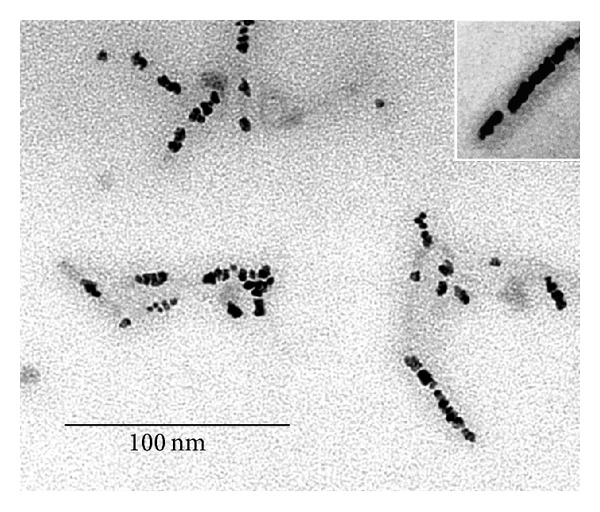
Nanoparticles of platinum fill the inner canal of the TMV VLP. TEM images of Pt nanoparticles in the inner canal of the virus-like rods produced from the coat protein of TMV. Incubation of VLP with (dien)platinum followed by electroless deposition of Pt atoms took place under the same conditions as with TMV. Inset in the upper corner indicates clearly the coat protein tube around the Pt nanoparticles. No contrasting of specimen was done.

It was discovered that (dien)platinum and clusters of the reduced platinum atoms do not bind to the VLP via the polar mode; instead, they fill the central hole of the TMV-like particles throughout their length (Figure 6). This finding supports the conclusion on the high sensitivity of (dien)platinum and/or Pt-NPs to spatial organization of functional chemical groups of CP on the surface of VLP and TMV.

### 3.5. Reduction of (Dien)platinum with Hypophosphite

**Figure 7:**
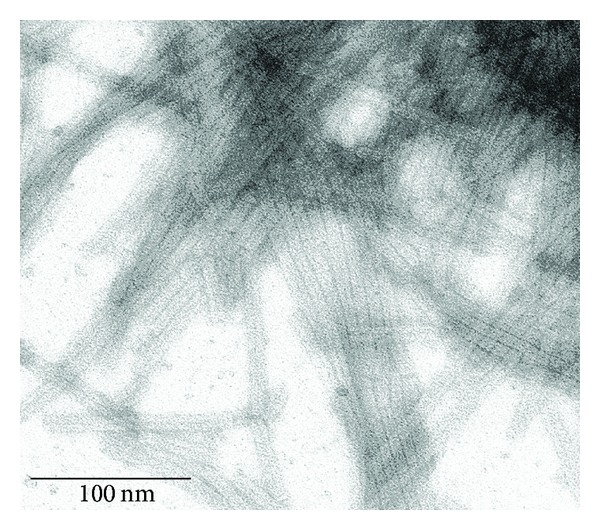
Chemical reduction of (dien)platinum on the TMV platform by hypophosphite. No additional staining was carried out.

Influence of the reduction agent and the reaction conditions were of much importance to understand the mechanism of the (dien)platinum interaction with TMV. Both, the surface and central axial hole of TMV were stained slightly with tiny atom clusters of platinum when (dien)platinum was reduced by 30 mM hypophosphite at pH 6-7 (Figure 7). Thus, modification of the reaction conditions drastically changed character of (dien)platinum-TMV interaction. 

Summing up, one can conclude that platinum coordinate compounds (dien)platinum and tetrachloroplatinate(IV) interact very selectively with spiral viruses TMV and PVX and after chemical reduction with borohydrides form nanoparticles via the polar mode with the 5′ end of the viruses (corresponding to RNA), as demonstrated through the polar uncoating of TMV with urea. 

Interestingly Balci et al. [4] described the self-assembling of prepared preliminary gold nanoparticles with a diameter of 6 nm and TMV into a metal-virus nanodumbbells. The gold nanoparticles were selectively bound to both ends of the virus rods and can be enlarged by electroless deposition of gold associated with shortening of the virus particles to yield gold-virus-gold dumbbells. 

Complexes of TMV and different ionic metals (Ni, Co, Cu, Fe, Ag, and Au) were examined by us as well. We found that copper could only interact with TMV in a polar mode, 100 TMV particles were examined and found 53% one end- and 1% two end- labeled TMV particles (data not shown) after borohydride reduction. 

## 4. Conclusions and Proposal

Nanoparticles of platinum that grows by polar mode on TMV and PVX helical viruses were produced by the borohydrides reduction of platinum coordinate compounds. Size of the platinum NPs depends on conditions of the chemical reduction of platinum atoms on the virus. Electron microscopy examination of polar uncoating of TMV particles showed that the Pt-NPs are bound concurrently to the terminal protein subunits and the 5′ end of encapsidated TMV RNA. 

Above and beyond nanorods, nanowires and nanorings produced earlier on the plant viruses as templates, tobacco mosaic virus, and potato X virus particles in a complex with nanoparticles of platinum, which look like a push-pin with platinum head and virus needle, were obtained. Thus, in this work, a novel structure of tobacco mosaic virus, and potato X virus particles in a complex with nanoparticles of platinum, which demonstrates extraordinary specificity of the coordinate metal interaction with a complex polyvalent biological construction, were obtained. 

TMV is established as one of the best-investigated models of macromolecular organization in biology. Moreover, partially and completely TMV can be reconstituted from its RNA and coat protein. The process of the TMV reconstitution was studied kinetically [16]. Thus, population of the TMV nucleoproteins of definite size (length) containing 5′ pole (in respect to RNA) can be obtained. On the other hand, 3′-terminal ribose of RNA in these nucleoproteins might be modified (oxidized, e.g.) and fixed covalently on any appropriate surface. One can propose that modified with platinum (or copper) population of these nanosize nucleoproteins of different lengths could serve as comb-like structures, or as strings of different thickness in a stringed instrument that are structurally (and functionally) similar to a musical organ. From our point of view, such wellorganized nanoparticle structures are of high demand in nanoelectronics. 

 We also cannot exclude that manifold ligands might be linked to the TMV and PVX viruses due to high chemical coordinate potential of platinum atoms.
